# First molecular detection and phylogenetic characterization of herpesviruses in Sri Lankan bats

**DOI:** 10.1186/s12917-026-06070-x

**Published:** 2026-11-07

**Authors:** Thejanee Perera, Franziska Schwarz, Therese Muzeniek, Sahan Siriwardana, Beate Becker-Ziaja, Inoka Perera, Shiroma Handunnetti, Jagathpriya Weerasena, Gayani Premawansa, Sunil Premawansa, Wipula Yapa, Andreas Nitsche, Claudia Kohl

**Affiliations:** 1 Institute of Biochemistry, Molecular Biology and Biotechnology, University of Colombo, Colombo, 00300 Sri Lanka; 2 Robert Koch Institute, Centre for Biological Threats and Special Pathogens, Highly Pathogenic Viruses (ZBS1), Berlin, Germany; 3 Department of Zoology and Environment Sciences, Faculty of Science, University of Colombo, Colombo, Sri Lanka; 4 Centre for International Health Protection, Public Health Laboratory Support (ZIG 4), Robert Koch In-stitute, Berlin, Germany; 5 Colombo North Teaching Hospital, Ragama, Sri Lanka

**Keywords:** Chiroptera, Sri Lanka, Herpesviridae, Betaherpesvirinae, Gammaherpesvirinae, Phylogeny, Zoonotic viruses, Bat-borne viruses

## Abstract

**Background:**

Herpesviruses are highly diverse and widely distributed pathogens infecting a broad range of animal hosts, including bats. Despite this wide distribution, they are typically extremely species-specific in their host associations. To better understand herpesvirus diversity in Sri Lankan bats, we screened 391 oral swab samples collected from five different bat species inhabiting Wavulgalge cave, Wellawaya (Uva Province, Sri Lanka) during three sampling periods (March 2018, July 2018, and January 2019) for herpesvirus DNA. A nested PCR assay was used, targeting the DNA polymerase gene of Herpesviruses.

**Results:**

In total, 152 samples (38.87%) were tested positive, within five bat species (*Hipposideros speoris* (9/22), *Hipposideros lankadiva* (2/4), *Miniopterus phillipsi* (61/224), *Rousettus leschenaultii* (18/46), and *Rhinolophus rouxii* (62/95). Statistical analysis indicated no significant difference in overall detection rates among species (*p* > 0.05), whereas prevalence varied significantly across sampling periods (*p* < 0.05), suggesting temporal factors such as breeding cycles or roosting behavior may influence virus transmission. Phylogenetic analysis showed that all sequences formed a distinct Sri Lankan clade, separate from global bat herpesviruses, and were distributed into two groups corresponding to betaherpesviruses and gammaherpesviruses. Alphaherpesviruses were not detected.

**Conclusions:**

Notably, sequences did not cluster by host bat species, suggesting limited host specificity among the Sri Lankan bat species examined. The high prevalence across multiple species highlights the complex host–virus dynamics. Overall, this study provides the first characterization of herpesviruses in Sri Lankan bats, contributing to global knowledge of bat virology.

**Supplementary Information:**

The online version contains supplementary material available at https://doi.org/10.1186/s12917-026-06070-x.

## Background

Herpesviruses (family *Herpesviridae*) are large, linear, double-stranded (ds) DNA viruses that can infect a broad variety of animal hosts, including humans [1–3]. Characteristically, the capsid of herpesviruses has a diameter of 100–110 nm. The synthesis of viral DNA and assembly of capsids occurs in the nucleus [1]. They establish lifelong infections with periodic cycles of latency and reactivation and thus are highly successful from an evolutionary perspective [4]. Some herpesviruses have a wide cell host range, replicate efficiently and destroy the infected cells rapidly (i.e. simplex virus 1), others are described to have a narrow host range (i.e. herpes virus 6) [1]. The family is divided into three subfamilies—*alphaherpesvirinae*, *betaherpesvirinae*, and *gammaherpesvirinae*—according to their biological properties [5]. The allocation to distinct subfamilies is based on distinct features. *Alphaherpesvirinae*, have a variable host range and relatively slow replication cycles. *Betaherpesvirinae*, have a restricted host range with a long replication cycle. *Gammaherpesvirinae*, have a host range limited to the family or order to which the natural host belongs [1]. More than 100 herpesvirus species have been identified in vertebrates, which suggests their prolonged coevolution with the host [6].

Chiropteran bats have emerged as model hosts in virology due to their exceptional species diversity, unique ecology, and ability to exist in massive multispecies colonies [7]. These features favor viral persistence, viral multiplication, and interspecies transmission. While RNA viruses, like coronaviruses, lyssaviruses, and filoviruses, have received extensive investigation, bats harbor a wide diversity of DNA viruses too, including herpesviruses [8–11]. Betaherpesviruses and gammaherpesviruses both have been detected worldwide in bats without demonstrated pathogenetic effects [8–11]. Additionally, mortality in experimental mice infection models was observed [10, 12, 13]. Certain bat herpesviruses were also shown to grow in cell lines derived from humans, monkeys, cats, and pigs, which point towards their cross-species transmission potential [14–16]. Interestingly, sequences related to human herpesviruses have also been reported in bat populations through metagenomic screening in countries such as Croatia, China, and Mexico, although these findings remain preliminary [17–19].

Despite the growing global knowledge on bat-associated herpesviruses, their diversity in Sri Lankan bats remains unexplored, with no systematic molecular characterization conducted to date [20]. Insights into herpesvirus diversity in Sri Lankan bats is valuable for understanding phylogeny, global ecology, virus–host co-evolution, and potential for cross-species transmission, even though their immediate zoonotic potential is considered low [2].

Herpesviruses in bats establish lifelong latent infections, likely persist in lymphoid or nervous tissues, and could therefore be undetectable by oral swabs during this dormant state [4]. However, this study provides a snapshot on potentially transmissible virus via saliva, though the real number of bats carrying herpes virus infection that cannot be detected by this approach remains unknown. Nested PCR increases sensitivity for detecting active or reactivated viruses in oral samples [11]. When combined with Sanger sequencing, this approach not only confirms herpesvirus infections but also offers initial insights into their genetic diversity and phylogenetic placement.

We present the first molecular evidence of herpesviruses in Sri Lankan bats. Oral swab samples were collected from five species roosting in Wavulgalge cave, one of the largest natural caves in Sri Lanka, on three occasions. Nested PCR targeting conserved herpesvirus genes, Sanger sequencing, and phylogenetic analysis were used to determine prevalence, genetic diversity, and evolutionary relationships of bat herpesviruses within this roost. Through phylogenetic characterization of detected sequences, this study fills a critical knowledge gap in South Asian bat virology and offers new insights into the ecology of herpesviruses in wildlife.

## Methods

The study was conducted with wild caught bats from Wavulgalge cave from Sri Lanka. The specimen collection was non-invasive (oral swabs). The bats were released after sample collection. No animals were euthanized or sacrificed. No anesthesia was necessary during sampling.

### Sampling location

**Fig. 1:**
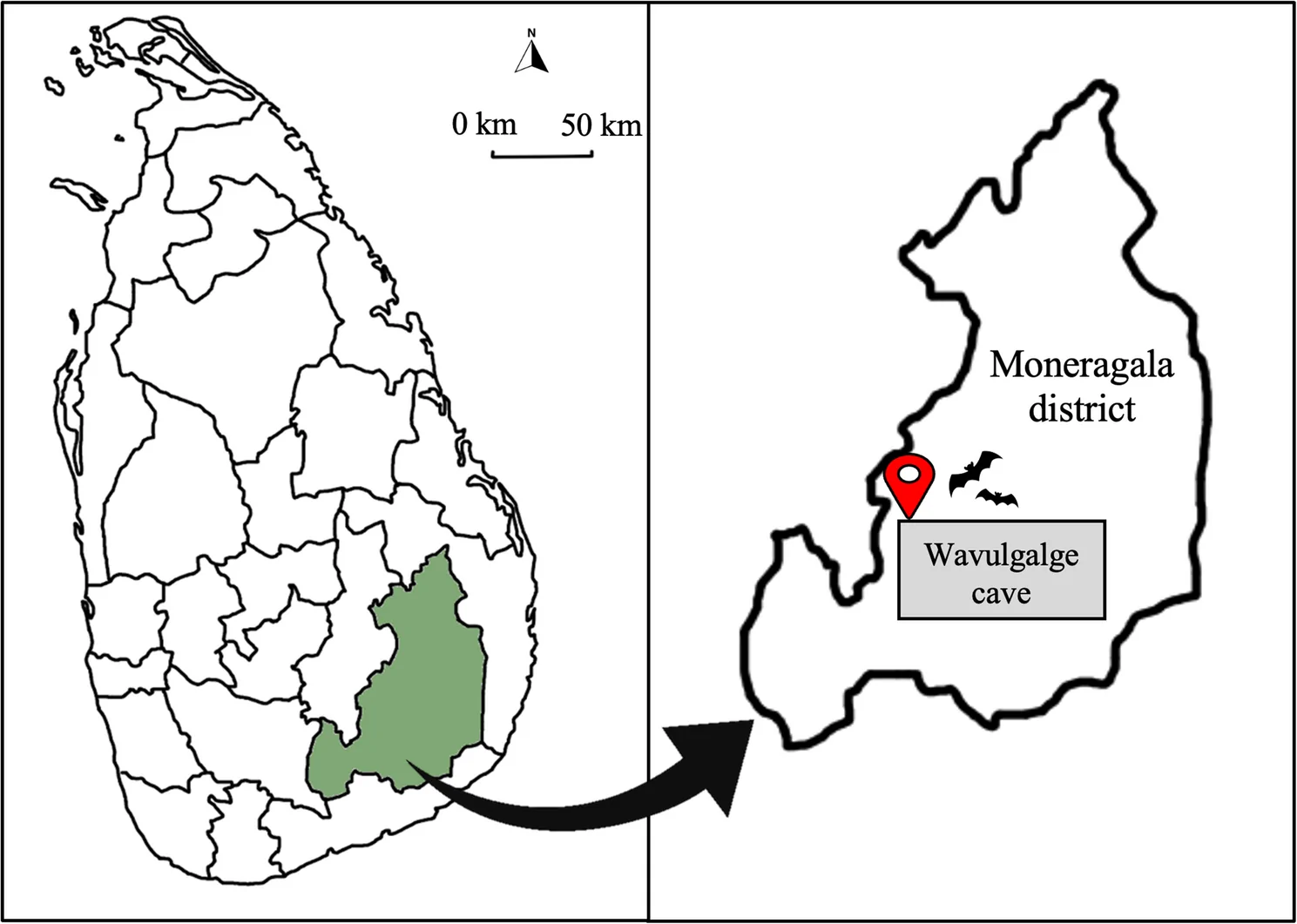
Sampling location: Wavulgalge cave, Sri Lanka. The location pin of the figure indicates the Wavulgalge cave, situated in Moneragala district, Sri Lanka. Copyright at 2025 Thejanee Perera

The study was conducted at Wavulgalge cave located in Koslanda, Monaragala district, Sri Lanka (6° 43’ 37.1676’’ N, 81° 3’11.7216’’ E) as shown in Fig. 1.

The cave is in a rural setting, surrounded by scattered houses and extensive rice fields within a 200-meter radius. The nearest village comprises dispersed dwellings along the main road, extending for nearly one kilometer from the cave entrance. This site was selected as it is one of the largest bat roosting habitats in Sri Lanka, hosting multiple bat species within a single colony [21, 22].

### Sampling period

Bat sampling was carried out in March and July 2018, and again in January 2019 to capture potential seasonal variation in viral prevalence. Individuals were captured with hand nets during their emergence from the cave. Each bat was examined and recorded for age, sex, and forearm length. Age was classified into juvenile, sub-adult, or adult categories, while sex determination was based on external sexual characteristics. During bat sampling, external examination was performed to document visible clinical signs. The results for the individual bats (i.e. molecular species identification on cytochrtome B, forearm length, sex) and the detailed sampling procedure are described in separate publications and summarized for the respective bats in supplementary Table 2 [21, 22].

### Biological sample collection

Oral swab samples were collected with sterile swabs, from five bat species- *Miniopterus phillipsi*, *Hipposideros speoris*, *Hipposideros lankadiva*,* Rousettus leschenaultii*, and *Rhinolophus rouxii*, as previously described [21]. Oral swabs were stored in 2 ml microtubes and transported in Va-Q-Tcon cooling boxes (va-Q-tec Thermal Solutions GmbH, Germany) with − 80 °C cooling packs and a dry shipper VOYAGEUR (Air Liquide, Austria) containing absorbed liquid nitrogen, ensuring the cold chain was maintained throughout the journey.

### Ethical considerations and safety measures

The study followed ethical guidelines, with permits from the Department of Wildlife Conservation, Sri Lanka (permit No. WL/3/2/05/18), and ethical clearance from the Institute of Biology, Sri Lanka (ERC IOBSL 170 01 18). Each bat was carefully captured, restrained manually with gloves, and sampled swiftly to minimize stress before being released at the site of capture [21]. The animal study protocol was approved by the Department of Wildlife Conservation, Sri Lanka (permit No. WL/3/2/05/18) and the Ethics Committee of the Institute of Biology, Sri Lanka (ERC IOBSL 170 01 18); https://www.iobsl.org/ehitcal-review/ethical-review-committee. The study did not contain any human specimens. The animals were wild caught and not privately owned or owned by another institution / individual / farm. The laboratory analyses of the collected samples were conducted following the BSL-2 conditions at the Robert Koch Institute, Berlin, Germany, adhering to strict safety protocols to minimize any potential zoonotic risks associated with handling biological materials.

### Molecular analysis of bat samples

Oral swab samples from bats were processed for nucleic acid extraction using the Qiagen Viral RNA Extraction Kit (Qiagen, Hilden, Germany), with DNA digestion steps omitted, following the manufacturer’s protocol. This strategy was applied to enable detection of a wider range of pathogens, including RNA viruses.

Samples were screened for herpesviruses using the nested PCR method described by Ehlers et al., with the exception that Taq Polymerase from Invitrogen was used instead of the described AmpliTaq gold [23]. Primers used for the primary and secondary amplifications are described in Table 1.

**Table 1:** Primers used in the PCR amplification of herpesviruses

Primer name	Primer sequence (5’-3’)	Amplicon length (bp)	Reference
285as KG1 (as)	gTC TTg CTC ACC Ag(N/I) TC(N/I) AC(N/I) CCY TT	688 and 424	[23]
285s DFA (s)	gAY TTY gC(N/I) TAY CC		
285s ILK (s)	TCC Tgg ACA AgC AgC AR(N/I) YSg C(N/I) AA		
286as IYG (as)	CAC AgA gTC CgT RTC (N/I) CC RTA (N/I) AT	175	
286s TGV (s)	TgT AAC TCg CTC TAY gg(N/I) TTY AC(N/I) gg(N/I) gT		

Degenerated bases are denoted by N(for any nucleotide), I(for inosine), Y(C or T), R(A or G), and S(C or G) [23]. s, sense; as, antisense

The extracted nucleic acid was then subjected to PCR amplification by the Ehlers assay as stated in the Table 1, using the DFA, ILK and KG1 primers targeting the DNA Polymerase gene. Three microliters of the extracted DNA were added to a PCR master mix containing 7.35 µl of PCR water, 2.5 µl of 10x buffer, 1 µl of 50 mM MgCl_2_, 2 µl of 2.5mM dNTPs, 1.25 µl of Dimethyl sulfoxide (DMSO), 0.4 µl of Taq Polymerase (5 U/ µl) and 2.5 µl of 10 µM of each DFA (s), ILK (s) and KG1(as) primers (s, sense; as, antisense). The thermal cycling conditions were as follows: initial denaturation at 95 °C for 2 min, followed by 45 cycles of denaturation at 95 °C for 20 s, annealing at 46 °C for 30 s and elongation at 72 °C for 30 s followed by a general extension at 72 °C for 10 min.

For the Nested PCR step, 1 µl of the primary amplification product was added to the second PCR master mix containing 11.85 µl of PCR water, 2.5 µl of 10x buffer, 1 µl of 50 mM MgCl_2_, 2 µl of 2.5mM dNTPs, 1.25 µl of DMSO, 0.4 µl of Taq Polymerase (AmpliTaq Gold DNA polymerase Kit, Thermo Fisher Scientific) and 2.5 µl of 10 µM of each TGV (s) and IYG (as) primers. The thermal cycling conditions for the nested PCR were as follows: initial denaturation at 95 °C for 2 min, followed by 45 cycles of denaturation at 95 °C for 20 s, annealing at 47 °C for 20 s and elongation at 72 °C for 20 s followed by a general extension at 72 °C for 10 min.

To analyze the PCR products, 0.5 µl of 6 x loading buffer was added to 5 µl of sample and loaded onto a 1.5% agarose gel, containing 1.25 µl Serva DNA stain clear G (SERVA Electrophoresis GmbH). TrackIt™ 100 bp DNA ladder from Thermo Scientific was used as a reference. 150 µl 0.5 x TAE buffer (Thermo Fisher Scientific) was used with 7.5 µl Serva DNA stain clear G. The gel was run for 30 min at 90 V and analyzed with the BioRad Gel Documentation system. PCR products from positive bands were purified using MSB Spin PCRapace purification kit (STRATEC, Birkenfeld, Germany) following the manufacturer’s instructions, to remove the excess primers and dNTPs.

### Sanger sequencing of the PCR amplicons

Purified PCR products were sequenced using the Sanger sequencing method. The amplicons were sequenced in both directions using the Big Dye Terminator cycle sequencing Kit (Applied Biosystems, Foster City, USA). An amount of 1 µl of the purified PCR product was added to the Sanger sequencing master mix containing 3 µl of PCR water 1.5 µl of 5× PCR buffer, 2 µl of BigDye 3.1 and 0.5 µl of 10 µM of respective forward or reverse primer. The thermal cycling conditions were as follows: initial denaturation at 96 °C for 2 min, followed by 25 cycles of denaturation at 96 °C for 10 s, annealing at 55 °C for 5 s, and extension at 60 °C for 4 min. Sequencing was performed using the Applied Biosystems 3500 Dx Genetic Analyzer, by the sequencing lab at Robert Koch Institute, Germany.

### Statistical and phylogenetic analysis of the sequences

The prevalence of herpesvirus infection among bats was analyzed using binomial logistic regression, with infection status as the response variable. Bat species and sampling month were included as predictor variables to evaluate their effects on infection odds. Logistic regression was chosen because the outcome is binary, and sample sizes were unbalanced across species and time points.

Analyses were performed in Python (stats models v0.14.0). Odds ratios (ORs) and 95% confidence intervals (CIs) were calculated from the regression coefficients. Statistical significance was assessed at *p* < 0.05. For small sample sizes, Fisher’s exact test was considered. Results are presented as ORs with corresponding 95% CIs and p-values, allowing interpretation of both the magnitude and significance of effects.

The nucleotide sequence analyses were performed using Geneious Prime^®^ 2023.1.2 and MEGA X. FASTA sequences were trimmed by the trim function of Geneious Prime^®^ 2023.1.2 to remove primer regions and assembled using the *De Novo Assembly* function to generate consensus sequences. These consensus sequences were compared with those in the GenBank database using the BLASTn tool. Sequences were aligned using the MUSCLE alignment tool in MEGA X. For phylogenetic reconstruction, a Maximum Likelihood (ML) tree was generated in MEGA X using the Kimura 2-parameter model with a gamma distribution (K2 + G). Tree robustness was evaluated by bootstrap analysis with 10,000 replicates, and the tree was rooted with the *Human herpesvirus 4* (NC_009334) sequence as an outgroup, model selection was based on the best fitting model test as described before [22].

### Sequence similarity analysis

To assess the genetic relatedness of the herpesvirus sequences obtained in this study, all 34 nucleotide sequences included in the phylogeentic analysis were aligned and subjected to pairwise similarity analysis. Percent identities were calculated for every sequence pair using global alignment (Needleman–Wunsch algorithm) implemented in Biopython (v1.85). The resulting similarity matrix was visualized as a heatmap using the Seaborn (v0.13.2) library in Python.

## Results

### Visible examination of bats

**Fig. 2:**
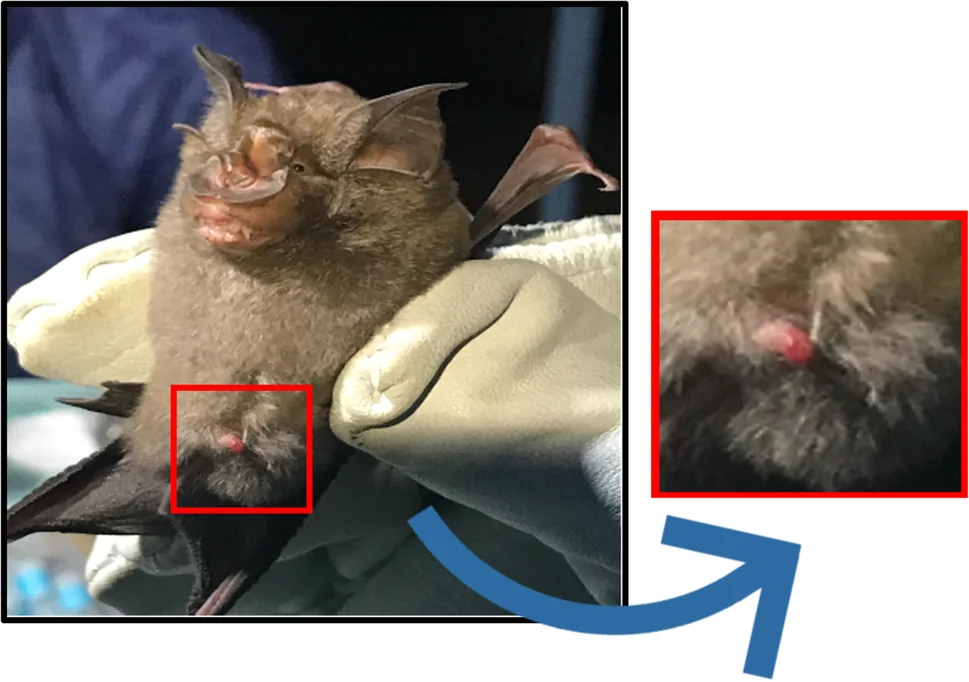
Observation of penile redness in a male R. rouxii bat (O386). Copyright at 2025 Thejanee Perera

A male *R. rouxii* (*O386*) exhibited penile redness during clinical examination as shown in Fig. 2. Additionally, two female *R. leschenaultii* bats (*O090*,* O358*) showed noticeable skin lesions and a male *Miniopterus* bat (*O354*) had two swellings near the chin. Subsequent testing confirmed the presence of bat herpesviruses in all four bats. However, there is no proof that the clinical signs are somehow related to herpesviruses.

### Screening of bat oral swab samples for herpesviruses using nested PCR method

Among the 391 bat oral swab samples tested, 152 (38.87%) yielded positive results, indicating the presence of herpesviruses in the Wavulgalge bat population.

Betaherpesvirus predominated, followed by gammaherpesvirus, with marked variation among species and sampling periods (Table 2).

**Table 2:** Detection of herpesvirus DNA in Sri Lankan bats by species, sampling period, and subfamily

Bat species	Mar 2018 *n*/*N* (%)	July 2018 *n*/*N* (%)	Jan 2019 *n*/*N* (%)	Total
*H. speoris*	1/2 (50.0)	1/1 (100.0)	7/19 (36.9)	9/22 (40.9)
*H. lankadiva*	–	–	2/4 (50.0)	2/4 (50.0)
*M. phillipsi*	4/6 (66.7)	40/188 (21.3)	17/30 (56.7)	61/224 (27.2)
*R. leschenaultii*	9/13 (69.2)	2/11 (18.2)	7/22 (31.8)	18/46 (39.1)
*R. rouxii*	45/63 (71.4)	0/0 (0)	17/32 (53.1)	62/95 (65.3)
Total	59/84 (70.2)	43/200 (21.5)	50/107 (46.7)	152/391 (38.9)

*N*  total samples, *n*  positive samples, (*%*) percent positive

### Diversity of herpesviruses among Wavulgalge bat population

Screening revealed the presence of both betaherpesviruses and gammaherpesviruses among bats inhabiting Wavulgalge cave, while no evidence of alphaherpesviruses was found in the population. These identified viruses were closely related to the herpesviruses that have been previously documented in bats from various other countries such as French Guiana, Martinique and Philippines [04.05.2025, NCBI, Genebank]. These positive results spanned across all the five bat species that were included in this study, as indicated in the Supplementary Table 1.

### Statistical and phylogenetic analysis of the sequences

Binomial logistic regression was used to assess the effects of bat species and sampling month on herpesvirus infection. The analysis revealed that bat species did not significantly influence detection odds (all *p* > 0.6). In contrast, sampling month was a significant predictor, with bats sampled in March 2018 exhibiting ~ 9-fold higher odds of infection (OR = 9.22, 95% CI: 3.79–22.38, *p* < 0.001) and those sampled in January 2019 showing ~ 3.8-fold higher odds (OR = 3.75, 95% CI: 1.87–7.53, *p* < 0.001) relative to July 2018. These results indicate that temporal variation, rather than host species, largely drives herpesvirus prevalence in the Wavulgalge cave bat population. The logistic regression results (odds ratios with 95% CIs and p-values) are depicted in Table 3 (H. lankadiva was not included in theis analysis due to sample numbers).

**Table 3:** Logistic regression – odds ratios for herpesvirus positivity

Predictor	Odd ratio (OR)	95% CI value	*p*-value
Species: *H. speoris*	0.67	0.08–5.69	0.71
Species: *M. phillipsi*	1.06	0.13–8.28	0.96
Species: *R. leschenaultii*	0.63	0.08–5.02	0.66
Species: *R. rouxii*	1.06	0.14–8.24	0.95
Month: March 2018	9.22	3.79–22.38	< 0.001
Month: January 2019	3.75	1.87–7.53	< 0.001

The month with the highest number of samples collected (July 2018) was used as the reference category in this logistic regression. H. lankadiva was not included in theis analysis due to sample numbers

**Fig. 3:**
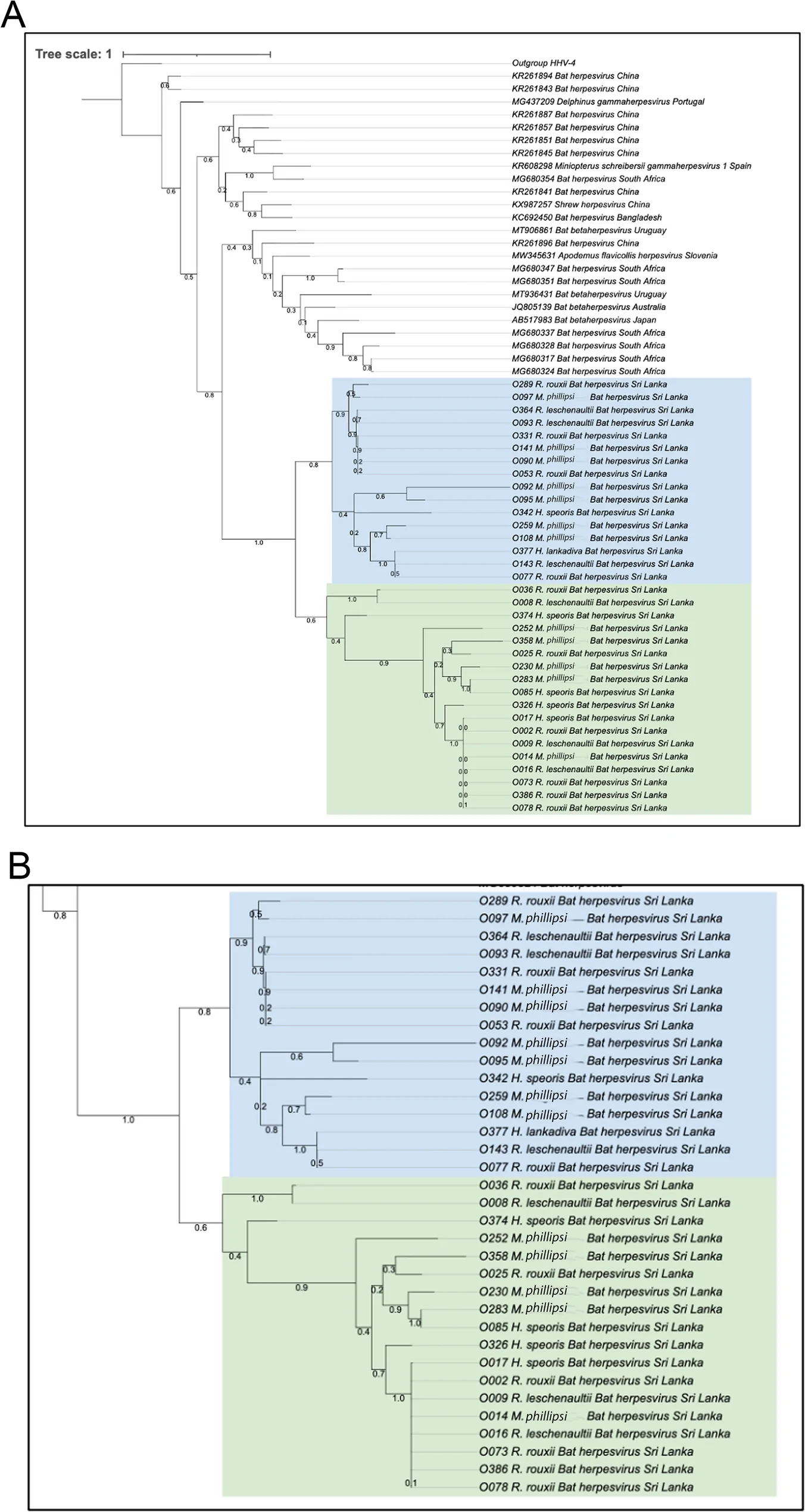
**a** Maximum Likelihood tree of herpesvirus DPOL gene (175 bp) sequences. The evolutionary history was inferred by using the Maximum Likelihood method and Kimura 2-parameter model [24]. The tree with the highest log likelihood (-7775.65) is shown. The bootstrap values are shown next to the branches. Initial tree(s) for the heuristic search were obtained automatically by applying Neighbor-Join and BioNJ algorithms to a matrix of pairwise distances estimated using the Maximum Composite Likelihood (MCL) approach, and then selecting the topology with superior log likelihood value. A discrete Gamma distribution was used to model evolutionary rate differences among sites (5 categories (+ G, parameter = 2.0667)). The tree is drawn to scale, with branch lengths measured in the number of substitutions per site. This analysis involved 51 nucleotide sequences of 175 bp length bp length. Codon positions included were 1st+2nd+3rd+Noncoding. There was a total of 556 positions in the final dataset. Evolutionary analyses were conducted in MEGA X [25]. The human herpesvirus 4 (NC_009334) was used as the outgroup. Sri Lankan bat herpesvirus clade. Sequences obtained in the present study are distinguished by a shaded background. A blue background highlights the betaherpesvirus clade, while a green background highlights the gammaherpesvirus clade. **b** Sri Lankan bat herpesvirus clade. Sequences obtained in the present study are distinguished by a shaded background. A blue background highlights the betaherpesvirus clade, while a green background highlights the gammaherpesvirus clade

Figure 3a and b displays the inferred phylogenetic tree. Sequences of herpesviruses utilized for constructing the phylogenetic tree are presented in the Supplementary Table 1. 36 of the 51 sequences obtained in this study were used for phylogenetic reconstruction as only unique sequences and further those with sufficient length and quality were further processed.

### Genetic similarity of detected herpesvirus sequences

**Fig. 4:**
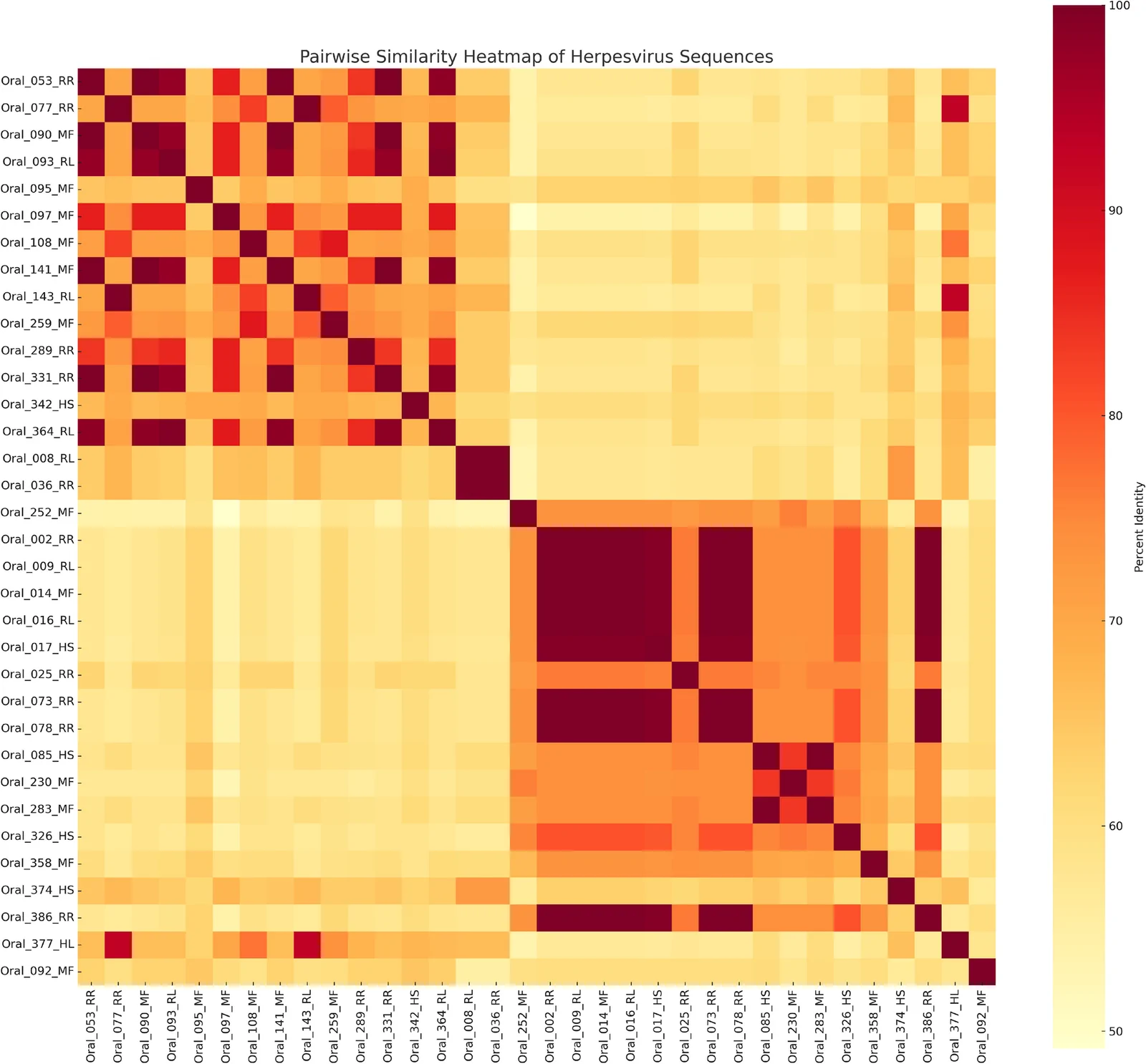
Heatmap of nucleotide sequence similarity (% identity) among Sri Lankan bat herpesviruses

Pairwise comparison revealed 51 unique herpesvirus sequences within the 152 positives (supplementary Table 1) with high overall genetic similarity, with percent identities ranging from 36.19% to 100% with a mean identity of 54.13%. The heatmap Fig. 4 illustrates clustering of Sri Lankan bat herpesvirus sequences based on 36 of the 51 unique nucleotide sequence with sufficient length. The individual date per bat (sex, age, forearm length is given in supplementary Table 2).

## Discussion

This study provides the first comprehensive overview of herpesvirus diversity, and phylogenetics in Sri Lankan bats occupying the Wavulgalge cave, Sri Lanka.

Nested PCR screening showed that 38.9% of bats were positive for herpesvirus DNA. Although raw detection rates varied among species and sampling periods, statistical analysis revealed no significant species-level differences. In contrast, detection rates differed significantly across sampling periods, suggesting that temporal factors such as breeding cycles, roosting behavior, or bat density may influence viral reactivation and shedding. *M. phillipsi* showed seasonal fluctuations due to migration, which may partly explain temporal variation in its samples. Nonetheless, since species was not a significant predictor overall, the observed monthly differences likely reflect true temporal dynamics in herpesvirus prevalence, with higher detection in March potentially resulting from seasonal viral reactivation or physiological stress in bats. These results align with previous studies indicating that bat herpesvirus infections can fluctuate over time, even if species-level differences are not statistically significant [26]. However, this study has pilot character and as no extensive ecological data on the species is available, this study is limited by comprising sex, age and reproduction cycles into the analysis. The detection of herpesviruses via oral swabs is inherently limited to periods of active viral shedding or reactivation, as these viruses establish lifelong latent infections in other host tissues [4, 27]. Consequently, a positive oral swab result likely reflects active or reactivated infection rather than total viral carriage. Thus, the number of bats tested positive may underestimate the real number of positive individuals. Furthermore, the study was constrained by a small sample size, particularly for *H. lankadiva*. Expanding sampling efforts and conducting longitudinal studies will be essential to better understand temporal trends, infection dynamics, and species-specific patterns in these bat populations. Additionally, limitations of the sampling method and the detection threshold of the assay should be considered when interpreting temporal fluctuations and host-specific patterns in our study.

Herpesvirus diversity in the Wavulgalge cave bat population was represented by viruses related to betaherpesviruses and gammaherpesviruses, while alphaherpesviruses were not detected. Multiple viral subfamilies were identified across all five bat species, may indicate that viral diversity is not strongly associated with host species within this sympatric roost. This pattern is in line with observations from other regions, including Central Africa and the Iberian Peninsula [8, 9, 11]. Phylogenetic analysis further supported these findings, revealing that viral sequences did not cluster according to host species. This suggests a lack of strict host specificity and a potential for cross-species transmission within the cave environment. Together, the prevalence, diversity, and phylogenetic patterns highlight that herpesvirus infections in this sympatric bat roost exhibit broad host ranges and high genetic diversity consistent with observations from previous studies [28]. For more robust and detailed phylogenetic analysis longer sequences would be beneficial and should be obtained to learn more on the exact allocation within the phylogenetic tree.

The Sri Lankan herpesvirus sequences formed two well-supported clades corresponding to betaherpesviruses and gammaherpesviruses, distinct from herpesviruses identified in bats from other countries such as China, Spain, South Africa, Bangladesh, Uruguay, Australia and Japan. This phylogenetic distinction may reflect geographic isolation, evolutionary divergence, or local adaptation of viruses to sympatric bat communities in Sri Lanka.

The high similarity (> 95%) between certain sequences as seen in Heatmap and maximum likelihood tree such as O002, O009, O014, and O017 identified from *R. rouxii*,* R. leschenaultii*,* M. phillipsi*,* H. speoris* bat species respectively suggest possibility of recent transmission events of an identical viral strain indicating a very close relationship between those bats, possibly from direct contact, due to sharing of roosting sites. Additionally, the moderate to low similarity values (< 50) likely represent different strains of the same virus that have been circulating and evolving within the bat population for an extended period. Overall, the combination of highly similar sequences and highly divergent ones paints a picture of a dynamic viral landscape within the Wavulgalge bat colony, with new strains being introduced or evolving over time.

Clinical observations in 4 of the 152 herpes PCR-positive bats—penile redness in *R. rouxii* and skin lesions in other species—highlight the complexity of interpreting visible signs in wild bat populations. Betaherpesviruses and gammaherpesviruses in bats are often asymptomatic, and herpesvirus-induced lesions in bats are poorly characterized. Consequently, it is not possible to attribute these lesions directly to herpesvirus infection based solely on oral swab PCR. Alternative explanations, including trauma, bacterial or fungal infection, parasites, or other viral agents (i.e. poxviruses), must also be considered. Establishing causality would require testing lesion-specific samples, combined with histopathology to identify viral cytopathic effects. These findings underscore the need for cautious interpretation of clinical signs in wild bats and the importance of complementary molecular and histological analyses. A possible association of clinical signs in bats and herpesvirus infections has yet to be elucidated.

## Conclusion

Overall, the combination of molecular screening, and phylogenetic analysis highlight the broad host range and genetic diversity of herpesviruses in Sri Lankan bats. These findings add to the growing body of evidence that bats harbor diverse herpesviruses, providing valuable insight into their ecology and viral diversity, and contributing to global understanding of bat-associated viruses.

## Supplementary Information

Supplementary Material 1.

Supplementary Material 2.

## Data Availability

The data presented in this study are openly available in GenBank (https://www.ncbi.nlm.nih.gov/genbank) database.
