# Supplementary material for: First molecular detection and phylogenetic characterization of herpesviruses in Sri Lankan bats
Source: BMC Vet Res. 2026 Nov 7;22:674. doi: 10.1186/s12917-026-06070-x (PMC13746721; doi:10.1186/s12917-026-06070-x)
Supplement: Supplementary file 1 [file 12917_2026_6070_MOESM1_ESM.docx]

| **Supplementary Table 1. BLAST results for the sequences obtained from the study.** | | | | | | |
| --- | --- | --- | --- | --- | --- | --- |
| **From the study** | | **BLAST result** | | | | |
| **Bat ID** | **Bat species**  **(molecular)** | **% ID** | **Herpes family** | **Accession number** | **Description** | **Host** |
| O_377 | *H. lankadiva* | 83.3% | Bat betaherpesvirus | AB517983 | Bat betaherpesvirus 2 gB, DPOL genes for glycoprotein B, DNA polymerase, complete and partial cds |  |
| O_017 | *H. speoris* | 80.00% | Bat herpesvirus | KR261887.1 | Bat herpesvirus isolate RB/13YF84 DNA polymerase gene, partial cds | *Rhinolophus blythi* |
| O_085 | *H. speoris* | 76.82% | Bat herpesvirus | KR261841.1 | Bat herpesvirus isolate HL/11HN1 DNA polymerase gene, partial cds | *Hipposideros larvatus* |
| O_309 | *H. speoris* | 80.37% | Bat herpesvirus | KR261887.1 | Bat herpesvirus isolate RB/13YF84 DNA polymerase gene, partial cds | *Rhinolophus blythi* |
| O_326 | *H. speoris* | 84.02% | Bat herpesvirus | KR261845.1 | Bat herpesvirus isolate HP/11HN110 DNA polymerase gene, partial cds | *Hipposideros pomona* |
| O_342 | *H. speoris* | 77.19% | Betaherpesvirus | MT906861.1 | Betaherpesvirinae sp. isolate 31 polymerase gene, partial cds |  |
| O_374 | *H. speoris* | 73.09% | Gammaherpesvirus | MG437209.1 | Gammaherpesvirinae sp. isolate DD-230-2012 DNA polymerase (DNApol) gene, partial cds |  |
| O_014 | *M. phillipsi* | 80.90% | Bat herpesvirus | KR261887 | Bat herpesvirus isolate RB/13YF84 DNA polymerase gene, partial cds | *Rhinolophus blythi* |
| O_065 | *M. phillipsi* | 71.20% | Bat herpesvirus | KR261857 | Bat herpesvirus isolate RB/13YF84 DNA polymerase gene, partial cds | *Rhinolophus blythi* |
| O_089 | *M. phillipsi* | 83.30% | Bat betaherpesvirus | AB517983 | Bat betaherpesvirus 2 gB, DPOL genes for glycoprotein B, DNA polymerase, complete and partial cds |  |
| O_090 | *M. phillipsi* | 83.50% | Bat herpesvirus | MG680317 | Bat herpesvirus isolate UP5110 DNA polymerase (pol) gene, partial cds | *Miniopterus natalensis* |
| O_092 | *M. phillipsi* | 82.60% | Bat herpesvirus | KR261896 | Bat herpesvirus isolate CS/14GZ13 DNA polymerase gene, partial cds | *Cynopterus sphinx* |
| O_095 | *M. phillipsi* | 77.30% | Mice betaherpesvirus | MW345631 | *Apodemus flavicollis* herpesvirus strain G12/19 DNA-dependent DNA polymerase gene, partial cds | *Apodemus flavicollis* |
| O_097 | *M. phillipsi* | 88.60% | Bat herpesvirus | MG680328 | Bat herpesvirus isolate UP5647 DNA polymerase (pol) gene, partial cds | *Rousettus aegyptiacus* |
| O_108 | *M. phillipsi* | 91.90% | Bat betaherpesvirus | AB517983 | Bat betaherpesvirus 2 gB, DPOL genes for glycoprotein B, DNA polymerase, complete and partial cds |  |
| O_141 | *M. phillipsi* | 83.50% | Bat herpesvirus | MG680317 | Bat herpesvirus isolate UP5110 DNA polymerase (pol) gene, partial cds | *Miniopterus natalensis* |
| O_153 | *M. phillipsi* | 84.70% | Bat herpesvirus | MG680347 | Bat herpesvirus isolate UP5701 DNA polymerase (pol) gene, partial cds | *Miniopterus natalensis* |
| O_186 | *M. phillipsi* | 84.70% | Bat herpesvirus | MG680351 | Bat herpesvirus isolate UP6344 DNA polymerase (pol) gene, partial cds | *Miniopterus natalensis* |
| O_206 | *M. phillipsi* | 77.30% | Mice betaherpesvirus | MW345631 | *Apodemus flavicollis* herpesvirus strain G12/19 DNA-dependent DNA polymerase gene, partial cds | *Apodemus flavicollis* |
| O_230 | *M. phillipsi* | 77.60% | Bat herpesvirus | KR261851 | Bat herpesvirus isolate MS/12HN28 DNA polymerase gene, partial cds | *Miniopterus schreibersii* |
| O_231 | *M. phillipsi* | 76.90% | Mice betaherpesvirus | MW345631 | *Apodemus flavicollis* herpesvirus strain G12/19 DNA-dependent DNA polymerase gene, partial cds | *Apodemus flavicollis* |
| O_252 | *M. phillipsi* | 83.80% | Gammaherpesvirus | KR608298 | *Miniopterus schreibersii* gammaherpesvirus 1 strain psc-28 DNA polymerase gene, partial cds | *Miniopterus schreibersii* |
| O_259 | *M. phillipsi* | 86.00% | Bat betaherpesvirus | JQ805139 | Bat betaherpesvirus B7D8, complete genome |  |
| O_269 | *M. phillipsi* | 83.80% | Gammaherpesvirus | KR608298 | *Miniopterus schreibersii* gammaherpesvirus 1 strain psc-28 DNA polymerase gene, partial cds | *Miniopterus schreibersii* |
| O_262 | *M. phillipsi* | 85.30% | Bat herpesvirus | MG680347 | Bat herpesvirus isolate UP5701 DNA polymerase (pol) gene, partial cds | *Miniopterus natalensis* |
| O_283 | *M. phillipsi* | 76.70% | Bat herpesvirus | KR261841 | Bat herpesvirus isolate HL/11HN1 DNA polymerase gene, partial cds | *Hipposideros larvatus* |
| O_291 | *M. phillipsi* | 83.30% | Bat betaherpesvirus | AB517983 | Bat betaherpesvirus 2 gB, DPOL genes for glycoprotein B, DNA polymerase, complete and partial cds |  |
| O_349 | *M. phillipsi* | 83.30% | Bat betaherpesvirus | AB517983 | Bat betaherpesvirus 2 gB, DPOL genes for glycoprotein B, DNA polymerase, complete and partial cds |  |
| O_358 | *M. phillipsi* | 70.80% | Shrew herpesvirus | KX987257 | Shrew herpesvirus isolate SM15GZBY378 DNA polymerase-like protein gene, partial cds |  |
| O_008 | *R. leschenaultii* | 88.50% | Bat herpesvirus | KR261843 | Bat herpesvirus isolate HP/11HN104 DNA polymerase gene, partial cds | *Hipposideros pomona* |
| O_009 | *R. leschenaultii* | 80.90% | Bat herpesvirus | KR261887 | Bat herpesvirus isolate RB/13YF84 DNA polymerase gene, partial cds | *Rhinolophus blythi* |
| O_016 | *R. leschenaultii* | 83.30% | Gammaherpesvirus | DQ789371 | *Crocuta crocuta* gammaherpesvirus 1 glycoprotein B (gB) and DNA polymerase (DPOL) genes, partial cds | *Crocuta crocuta* |
| O_033 | *R. leschenaultii* | 83.30% | Gammaherpesvirus | DQ789371 | *Crocuta crocuta* gammaherpesvirus 1 glycoprotein B (gB) and DNA polymerase (DPOL) genes, partial cds | *Crocuta crocuta* |
| O_093 | *R. leschenaultii* | 83.50% | Bat herpesvirus | MG680317 | Bat herpesvirus isolate UP5110 DNA polymerase (pol) gene, partial cds | *Miniopterus natalensis* |
| O_143 | *R. leschenaultii* | 85.30% | Bat betaherpesvirus | MT936431 | Bat betaherpesvirus isolate LMM9 polymerase gene, partial cds |  |
| O_299 | *R. leschenaultii* | 85.30% | Bat betaherpesvirus | MT936431 | Bat betaherpesvirus isolate LMM9 polymerase gene, partial cds |  |
| O_332 | *R. leschenaultii* | 85.30% | Bat betaherpesvirus | MT936431 | Bat betaherpesvirus isolate LMM9 polymerase gene, partial cds |  |
| O_364 | *R. leschenaultii* | 84.10% | Bat herpesvirus | MG680317 | Bat herpesvirus isolate UP5110 DNA polymerase (pol) gene, partial cds | *Miniopterus natalensis* |
| O_002 | *R. rouxii* | 80.80% | Bat herpesvirus | KR261887 | Bat herpesvirus isolate RB/13YF84 DNA polymerase gene, partial cds | *Rhinolophus blythi* |
| O_025 | *R. rouxii* | 79.10% | Unidentified herpesvirus | KC692450 | Unidentified herpesvirus isolate PgHV-3 DNA polymerase gene, partial cds |  |
| O_036 | *R. rouxii* | 88.50% | Bat herpesvirus | KR261843 | Bat herpesvirus isolate HP/11HN104 DNA polymerase gene, partial cds | *Hipposideros pomona* |
| O_053 | *R. rouxii* | 83.50% | Bat herpesvirus | MG680317 | Bat herpesvirus isolate UP5110 DNA polymerase (pol) gene, partial cds | *Miniopterus natalensis* |
| O_073 | *R. rouxii* | 80.90% | Bat herpesvirus | KR261887 | Bat herpesvirus isolate RB/13YF84 DNA polymerase gene, partial cds | *Rhinolophus blythi* |
| O_077 | *R. rouxii* | 83.30% | Bat betaherpesvirus | AB517983 | Bat betaherpesvirus 2 gB, DPOL genes for glycoprotein B, DNA polymerase, complete and partial cds |  |
| O_078 | *R. rouxii* | 83.30% | Gammaherpesvirus | DQ789371 | *Crocuta crocuta* gammaherpesvirus 1 glycoprotein B (gB) and DNA polymerase (DPOL) genes, partial cds | *Crocuta crocuta* |
| O_289 | *R. rouxii* | 84.20% | Bat herpesvirus | MG680324 | Bat herpesvirus isolate UP6420 DNA polymerase (pol) gene, partial cds | *Rousettus aegyptiacus* |
| O_298 | *R. rouxii* | 79.60% | Unidentified herpesvirus | KC692450 | Unidentified herpesvirus isolate PgHV-3 DNA polymerase gene, partial cds |  |
| O_319 | *R. rouxii* | 83.30% | Gammaherpesvirus | DQ789371 | *Crocuta crocuta* gammaherpesvirus 1 glycoprotein B (gB) and DNA polymerase (DPOL) genes, partial cds | *Crocuta crocuta* |
| O_331 | *R. rouxii* | 83.50% | Bat herpesvirus | MG680317 | Bat herpesvirus isolate UP5110 DNA polymerase (pol) gene, partial cds | *Miniopterus natalensis* |
| O_387 | *R. rouxii* | 82.40% | Gammaherpesvirus | DQ789369 | *Rupicapra rupicapra* gammaherpesvirus 1 glycoprotein B (gB) and DNA polymerase (DPOL) genes, partial cds | *Rupicapra rupicapra* |

Results from the study were compared with the sequences from the NCBI database using the BLAST tool. BLAST results for each sequence with their respective %ID, Herpesvirus with the highest percent ID and the harboring host species are summarized above. Color codes are as follows, *H. speoris: Yellow*, *H. lankadiva:Blue, M. phillipsi:Pink*, *R. leschenaultii:Green, R. rouxii:Purple*
