# Supplementary material for: First molecular detection and phylogenetic characterization of herpesviruses in Sri Lankan bats
Source: BMC Vet Res. 2026 Nov 7;22:674. doi: 10.1186/s12917-026-06070-x (PMC13746721; doi:10.1186/s12917-026-06070-x)
Supplement: Supplementary file 2 [file 12917_2026_6070_MOESM2_ESM.docx]

| **Bat Nr.** | **Species (molecular)** | **Age class** | **Sex** | **Forearm (cm)** |
| --- | --- | --- | --- | --- |
| 2 | ***R.rouxii*** |  |  |  |
| 8 | ***R.leschenaultii*** |  |  |  |
| 9 | ***R.leschenaultii*** |  |  |  |
| 14 | ***Human*** |  |  |  |
| 16 | ***R.leschenaultii*** |  |  |  |
| 17 | ***H.speoris*** |  |  |  |
| 25 | ***R.rouxii*** |  |  |  |
| 33 | ***R.leschenaultii*** |  |  | 4.50 |
| 36 | ***R.rouxii*** |  |  | 5.00 |
| 53 | ***R.rouxii*** |  | F | 7.50 |
| 65 | ***R.leschenaultii*** |  | F | 5.08 |
| 73 | ***R.rouxii*** | Adult | M | 4.94 |
| 77 | ***R.rouxii*** | Adult | F | 4.61 |
| 78 | ***R.rouxii*** |  |  | 4.93 |
| 85 | ***H.speoris*** | Adult | M | 4.76 |
| 89 | ***M.phillipsi*** |  | F | 4.64 |
| 90 | ***M.phillipsi*** | Sub-Adult | F | 5.67 |
| 92 | ***M.phillipsi*** | Adult | F | 7.92 |
| 93 | ***R.leschenaultii*** |  | M |  |
| 95 | ***M.phillipsi*** | Adult | F | 4.38 |
| 97 | ***M.phillipsi*** | Sub-Adult | M | 6.71 |
| 108 | ***M.phillipsi*** | Adult | F | 4.51 |
| 141 | ***M.phillipsi*** | Adult | F | 7.00 |
| 143 | ***R.leschenaultii*** | Adult | F | 4.75 |
| 153 | ***M.phillipsi*** | Adult | M | 4.59 |
| 186 | ***M.phillipsi*** | Adult | F | 4.70 |
| 206 | ***M.phillipsi*** | Adult | F | 4.35 |
| 230 | ***M.phillipsi*** | Adult | F | 4.75 |
| 231 | ***M.phillipsi*** | Adult | M | 4.76 |
| 252 | ***M.phillipsi*** | Adult | F | 4.31 |
| 259 | ***M.phillipsi*** | Adult | F | 4.32 |
| 262 | ***M.phillipsi*** | Adult | F | 4.61 |
| 269 | ***M.phillipsi*** |  |  |  |
| 283 | ***M.phillipsi*** | Adult | F | 4.50 |
| 289 | ***R.rouxii*** | Adult | F | 6.74 |
| 291 | ***M.phillipsi*** | Adult | M | 4.78 |
| 299 | ***R.leschenaultii*** | Adult | F | 4.53 |
| 309 | ***H.speoris*** | Adult | F | 4.95 |
| 319 | ***R.rouxii*** | Adult | F | 4.98 |
| 326 | ***H.speoris*** | Adult | M | 5.27 |
| 331 | ***R.rouxii*** | Juvenile | F | 6.75 |
| 332 | ***R.leschenaultii*** | Adult | M | 4.67 |
| 342 | ***H.speoris*** | Adult | M | 4.98 |
| 349 | ***M.phillipsi*** | Adult | M | 4.55 |
| 358 | ***M.phillipsi*** | Adult | F | 7.04 |
| 364 | ***R.leschenaultii*** | Adult | F | 6.79 |
| 374 | ***H.speoris*** | Adult | F | 8.78 |
| 377 | ***H.lankadiva*** | Adult | M | 4.43 |
| 387 | ***R.rouxii*** | Sub-Adult | M | 4.92 |

Supplementary table 2: Individual bat number, Species, Age class, Sex (F, female; M, male) and forearm length in cm are described where available.
